# Nitric Oxide Dismutase (*nod*) Genes as a Functional Marker for the Diversity and Phylogeny of Methane-Driven Oxygenic Denitrifiers

**DOI:** 10.3389/fmicb.2019.01577

**Published:** 2019-07-10

**Authors:** Baoli Zhu, Jiaqi Wang, Lauren M. Bradford, Katharina Ettwig, Baolan Hu, Tillmann Lueders

**Affiliations:** ^1^Institute of Groundwater Ecology, Helmholtz Zentrum München, Munich, Germany; ^2^Department of Microbiology, Radboud University Nijmegen, Nijmegen, Netherlands; ^3^Chair of Ecological Microbiology, Bayreuth Center of Ecology and Environmental Research (BayCEER), University of Bayreuth, Bayreuth, Germany; ^4^Department of Environmental Engineering, Zhejiang University, Hangzhou, China

**Keywords:** nitric oxide dismutation, oxygenic methanotrophs, oxygenic denitrification, NC10, *nod*, *pmoA*

## Abstract

Oxygenic denitrification represents a new route in reductive nitrogen turnover which differs from canonical denitrification in how nitric oxide (NO) is transformed into dinitrogen gas. Instead of NO reduction via N_2_O to N_2_, NO is proposed to be directly disproportionated into N_2_ and O_2_ in oxygenic denitrification, catalyzed by the putative NO dismutase (Nod). Although a high diversity of *nod* genes has been recovered from various environments, still little is known about the niche partitioning and ecophysiology of oxygenic denitrifiers. One constraint is that *nod* as a functional marker for oxygenic denitrifiers is not well established. To address this issue, we compared the diversity and phylogeny of *nod*, 16S rRNA and *pmoA* gene sequences of four NC10 enrichments that are capable of methane-driven oxygenic denitrification and one environmental sample. The phylogenies of *nod*, 16S rRNA and *pmoA* genes of these cultures were generally congruent. The diversity of NC10 bacteria inferred from different genes was also similar in each sample. A new set of NC10-specific *nod* primers was developed and used in qPCR. The abundance of NC10 bacteria inferred from *nod* genes was constantly lower than via 16S rRNA genes, but the difference was within one order of magnitude. These results suggest that *nod* is a suitable molecular marker for studying the diversity and phylogeny of methane-driven oxygenic denitrifiers, the further investigation of which may be of value to develop enhanced strategies for sustainable nitrogen or methane removal.

## Introduction

The defining step of oxygenic denitrification, the proposed nitric oxide (NO) dismutation, is conceived as an unusual oxygen-forming process, suggested to occur in nitrite-dependent anaerobic methanotrophs of the NC10 phylum (*e.g., Candidatus* Methylomirabilis oxyfera) and in a denitrifying alkane-degrading Gammaproteobacterium (Ettwig et al., 2010, 2012; Zedelius et al., 2011). In conventional denitrification, NO is reduced via N_2_O to N_2_ (Schreiber et al., 2012; Zhang et al., 2019). In contrast, in oxygenic denitrification, NO is suggested to be disproportionated directly into N_2_ and O_2_, thus bypassing the ozone-depleting potent greenhouse gas nitrous oxide (N_2_O). The released O_2_, in theory, could enable the use of aerobic catabolic routes in anoxic habitats, possibly entailing ecophysiological advantage for microbes to thrive on recalcitrant substrates in O_2_-limited environments (Zhu et al., 2017). So far, NO dismutation has been suggested to be involved in the oxidation of methane, alkanes (C6-C30) and benzene (Ettwig et al., 2010; Zedelius et al., 2011; Atashgahi et al., 2018). Recent molecular evidence, however, suggested that NO dismutation potentially occurs in only distantly related microorganisms with diverse catabolic capabilities (Zhu et al., 2017). Nonetheless, the current understanding of oxygenic denitrification, its key step of NO dismutation and the microbes hosting this capacity (referred to as oxygenic denitrifiers) is still very limited. So far, the NO dismutation step distinguishes, but not mutually excludes, oxygenic denitrifiers from conventional denitrifiers. It is known that the genes coding for enzymes involved in reductive N-cycling, e.g., *nif, nir*, and *nor*, are often subject to lateral gene transfer (Philippot, 2002; Jones et al., 2008; Andam et al., 2015). This complicates the inference of denitrifiers’ phylogeny via functional genes. Thus far, the possible evolutionary history of NO dismutase genes (*nod*) is unresolved. Although diverse *nod* gene pools have been recovered from the environment (Zhu et al., 2017; Zhang et al., 2018), their phylogenetic association often remains unclear.

Among all oxygenic denitrifiers, anaerobic methanotrophs of the NC10 phylum have been most intensively studied. They use CH_4_ as electron donor to fuel oxygenic denitrification (Ettwig et al., 2010). NC10 bacteria are present in various natural and engineered systems, such as wetlands, rivers, lakes, rice paddies, tidal zones, and wastewater treatment plants (Luesken et al., 2011b; Wang et al., 2012, 2015, 2018; Zhu et al., 2012; Shen et al., 2013, 2014; He et al., 2015). Due to their widespread occurrence and methane-oxidizing activity in nature, NC10 bacteria could actually represent an important methane sink in the environment (Zhu et al., 2012; Deutzmann et al., 2014; Hu et al., 2014). Still, the NC10 phylum has no pure culture representatives to date. Based on 16S rRNA sequence information, several groups within the phylum (group *a, b, c, d*, and *e*) have been classified (Ettwig et al., 2009; He et al., 2015). Of those, only members of the phylogroups *a* were found to be dominant in methane-oxidizing enrichment cultures obtained from various environments. The partial genome reconstruction of the clade *d* NC10 bacterium CSP1-5 from a subsurface sample suggested the capability for aerobic methanol oxidation and nitrate reduction, but not methane oxidation and NO dismutation (Hug et al., 2016). Thus, it is not likely that all members of the NC10 phylum are oxygenic methanotrophs.

It is known that 16S rRNA genes often do not allow inferring metabolic functions for environmental microbes. For aerobic methanotrophs, *pmoA* genes are a useful functional marker for diversity and phylogeny studies, and have been frequently used to target these populations in the environment (Bourne et al., 2001; Pacheco-Oliver et al., 2002). *pmoA* has also been used as a marker for methanotrophic NC10 bacteria, which also use particulate methane monooxygenase to activate methane. *M. oxyfera*-like *pmoA* sequences form a cluster distinct from *pmoA* of aerobic methanotrophs (Luesken et al., 2011c). When using a suggested OTU cut-off of 3 and 10% sequence similarity for 16S rRNA and *pmoA* (Luke and Frenzel, 2011), respectively, the diversity of NC10 *pmoA* genes recovered so far is much lower than the known NC10 16S rRNA gene diversity. Information about the presence of NC10 *nod* genes in different environments (Bhattacharjee et al., 2016; Padilla et al., 2016; Zhu et al., 2017) and in enrichment cultures is still scarce. This calls for a rigorous assessment of the utility and comparability of the different marker gene assays (*nod, pmoA*, and 16S) to recover the full spectrum of oxygenic methanotrophs within the NC10 phylum present in a given habitat.

In this study, the diversity and phylogeny of *nod* genes in four NC10 enrichment cultures and one aquifer sample were compared to the 16S rRNA and *pmoA* genes of the NC10 phylum detectable in the same samples. New NC10-specific *nod* primers were developed and validated, and NC10 abundance was evaluated by *nod* as well as 16S rRNA gene-targeted qPCR. Our results suggest that *nod* can be a functional and also a phylogenetic marker for oxygenic methanotrophs within the NC10 phylum. This will be useful for future studies seeking to address the ecology and ecophysiology of these enigmatic microbes in natural systems.

## Materials and Methods

### NC10 Enrichment Cultures and Aquifer Sample

NC10 bacteria have been previously enriched under denitrifying, methane-oxidizing conditions, with nitrite and/or nitrate as electron acceptors. Four sediment-free NC10 enrichment cultures maintained under different conditions were analyzed here (Table 1). In cultures AAA-NC10, DAMO-VI and DAMO-CANON, *M. oxyfera* related bacteria were dominant (Ettwig et al., 2009, 2016). In DAMO-SC, a different NC10 species, *M. sinica* was dominant (He et al., 2016). Aquifer sediment samples from Siklós were collected in April 2015 from the bottom of a monitoring well in the center of a xylene plume in Siklós, Hungary (Tancsics et al., 2013), where diverse *nod* sequences have been previously reported (Zhu et al., 2017).

**Table 1:** NC10 enrichment cultures and aquifer sample analyzed in this study.

Designation	Inoculum	Enrichment conditions		References	NC10 abundance (copy ng^-1^ DNA)^#^	
				
		Electron acceptor	Electron donor		16S rRNA	*nod*
AAA-NC10	NC10 enrichment settler biomass	nitrate	CH_4_	Ettwig et al., 2016	9.2^∗^10^4^	6.5^∗^10^4^
DAMO-VI	ditch sediment	nitrate and nitrite	CH_4_	Ettwig et al., 2009	6.4^∗^10^5^	4.8^∗^10^5^
DAMO-CANON	DAMO-VI reactor biomass	nitrite	CH_4_ and NH_3_	Luesken et al., 2011a	3.7^∗^10^5^	2.3^∗^10^5^
DAMO-SC	paddy soil	nitrite	CH_4_	He et al., 2016	7.2^∗^10^4^	2.5^∗^10^4^
Siklós aquifer		environmental sample	Xylene dominated hydrocarbons	Zhu et al., 2017	3.0^∗^10^3^	8.3^∗^10^2^


*# average of two different qPCR assays, each including three replications. The abundance of NC10 bacteria was inferred from both 16S rRNA and *nod* genes.*

### Dna Isolation

DNA of Siklós aquifer sediment was isolated as previously described (Pilloni et al., 2012) with minor modification, the final DNA precipitation was done at 4°C instead of 20°C. For DNA isolation from enrichment cultures, 0.5 – 1.0 ml homogenized culture was pipetted into 1.5 ml Eppendorf tubes, which were spun at 13,000 rpm for 2 min. The supernatant was then removed and the remaining biomass was weighed. DNA was isolated with the Power Soil DNA isolation kit (MoBio Laboratories Inc., United States) according to the manufacturer’s protocol. DNA concentration and quality were checked with the Quant-iT PicoGreen dsDNA Assay Kit (Thermo Fisher, Waltham, United States) on a MX3000p cycler (Agilent, Santa Clara, United States) and by standard agarose gel electrophoresis.

### NC10-Specific *nod* Primers

First, the general *nod* primer pair nod684Fv2 and nod1706Rv2 (Zhu et al., 2017) was used to amplify *nod* gene fragments from NC10 enrichment cultures. The amplicons were cloned and sequenced as previously described (Winderl et al., 2008). The resulting *nod* sequences and previously reported NC10 cluster *nod* sequences (Zhu et al., 2017) were aligned with reference *nod* sequences in MEGA6 (Tamura et al., 2013). Based on the updated alignment, a new set of NC10-specific *nod* gene primers (nod840F and nod1012R) was developed, which should strictly target a 173 bp fragment of *nod* sequences within the NC10 phylum and be optimally suited for qPCR (Table 2). The specificity of this primer pair was verified by sequencing the short amplicons using environmental DNA extracted from Siklós aquifer sediment as template. The primer pair was used for qPCR analysis.

**Table 2:** primers used for cloning and qPCR analysis in this study.

Primer	Sequence (5′– >3′)	Target gene	Application	Amplicon size (bp)^∗^	References
193F	GAC CAA AGG GGG CGA GCG	NC10 16S rRNA	cloning	859	Ettwig et al., 2009
1027R	TCT CCA CGC TCC CTT GCG				
qP1F	GGG CTT GAC ATC CCA CGA ACC TG	NC10 16S rRNA	qPCR	201	Ettwig et al., 2009
qP1R	CGC CTT CCT CCA GCT TGA CGC				
nod684Fv2	STAYACHCAYAACTGGCC	general *nod*	cloning	1023	Zhu et al., 2017
nod1706Rv2	GGCTTSGCRATCCAGTAGAAG				
nod840F	CAAYGGSCGSGAYTGGTCRC	NC10 *nod*	qPCR	173	This study
nod1012R	CHGGWCCNCCRCCRAYRAARTC				
A189b	GGNGACTGGGACTTYTGG	NC10 *pmoA*	cloning	534	Luesken et al., 2011c
cmo682	AAAYCCGGCRAAGAACGA				


*^∗^Based on *Methylomirabilis oxyfera* (FP565575) 16S rRNA and *nod* (DAMO_2437) gene sequences.*

### PCR and qPCR

DNA samples diluted by 10- or 100-fold were used as template for *nod* gene PCR analysis. Primer pairs used are listed in Table 2. To recover a potentially increased diversity for all analyzed *nod, pmoA*, and 16S rRNA genes, gradient PCRs with the following cycling conditions were performed: a 3 min initial dissociation at 96°C, followed by 30 cycles of amplification (45 s at 95°C, 60 s at 52–62°C, and 90 s at 72°C), and a final 5 min extension at 72°C. All PCRs were performed in 25 μl reactions containing nuclease-free H_2_O, 1 × PCR buffer, 1.5 mM MgCl_2_, 0.1 mM dNTPs, 0.5 U Taq polymerase (all Fermentas GmBH, Basel, Switzerland), 5 μg BSA (Roche Diagnostics GmbH, Basel, Switzerland), 0.5 μM of each primer, and 1 μl template DNA. PCR products were checked by standard agarose gel electrophoresis.

NC10 abundance in each sample was quantified by qPCR targeting *nod* as well as 16S rRNA genes, using the NC10-specific *nod* primers nod840F/nod1012R and the NC10-specific 16S rRNA primers qP1F/qP1R, respectively (Table 2). pGEM-T-easy vectors with respective inserts were used as standards. Sample DNA was quantified in 10- and 100-fold dilutions. Standards and samples were quantified in triplicate qPCR reactions. qPCR reactions (25 μl volume) were carried out with MX3000p cycler (Agilent, Santa Clara, United States). 2 × Takyon SYBR master mix (Eurogentec, Cologne, Germany) with Rox as the reference dye. The annealing temperatures used for *nod* and 16S rRNA qPCR were 57°C and 62°C, respectively. qPCR analyses with efficiencies of 100 ± 10% were used for calculating gene abundances. Absolute *nod* and 16S rRNA gene abundance of each sample were calculated per ng of genomic DNA.

### Cloning and Phylogenetic Analysis

*nod, pmoA*, and 16S rRNA PCR products of different annealing temperatures from each sample were pooled and purified with PCRextract spin columns (5Prime, Hamburg, Germany) according to the manufacturer’s protocol. Purified PCR products were ligated into the pGEM-T Easy Vector (Promega, Madison, United States) and then transformed into *Escherichia coli* JM109 competent cells. Selection of clones with correct insert size and Sanger sequencing was done as previously described (Winderl et al., 2008). High-quality sequences obtained were aligned with selected respective reference sequences with the ClustalW algorithm with default settings and were visually checked and adjusted before phylogeny constructions. *nod* and *pmoA* sequences were aligned with their translated protein sequences. Phylogenetic trees were constructed with MEGA6 using the neighbor-joining method. The robustness of each tree topology was tested by bootstrap analysis (1,000 replicates).

### Sequence Deposition

Representative 16S rRNA, *pmoA* and *nod* gene sequences obtained in this study were deposited at NCBI under the accession numbers MK909154 – MK909155, MK909156 – MK909157, and MK909158 – MK909164, respectively.

## Results and Discussion

### NC10 16S rRNA Gene Diversity in Enrichment Cultures and Aquifer Sediment

**FIGURE 1:**
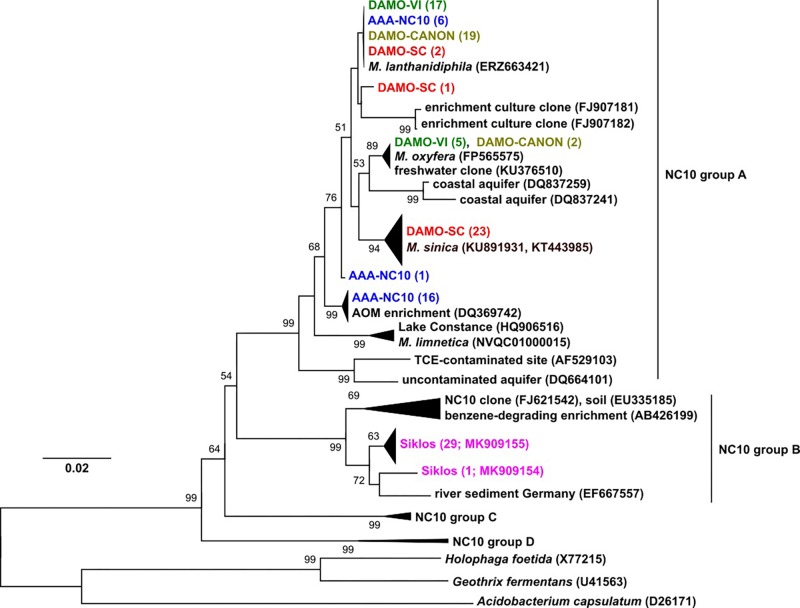
The 16S rRNA phylogeny of NC10 bacteria in the four reactors and Siklós aquifer. Sequences from different samples are labeled in different colors and are consistent in all figures. The number in bracket indicates number of clones in the branch or accession number of the reference and representative sequence.

**FIGURE 2:**
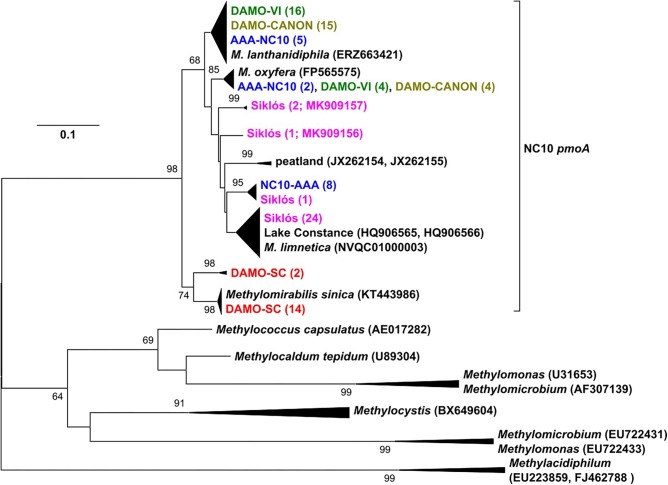
The *pmoA* phylogeny of NC10 bacteria in the four reactors and Siklós aquifer. Sequences from different samples are labeled in different colors and are consistent in all figures. The number in bracket indicates number of clones in the branch or accession number of the reference and representative sequence.

**FIGURE 3:**
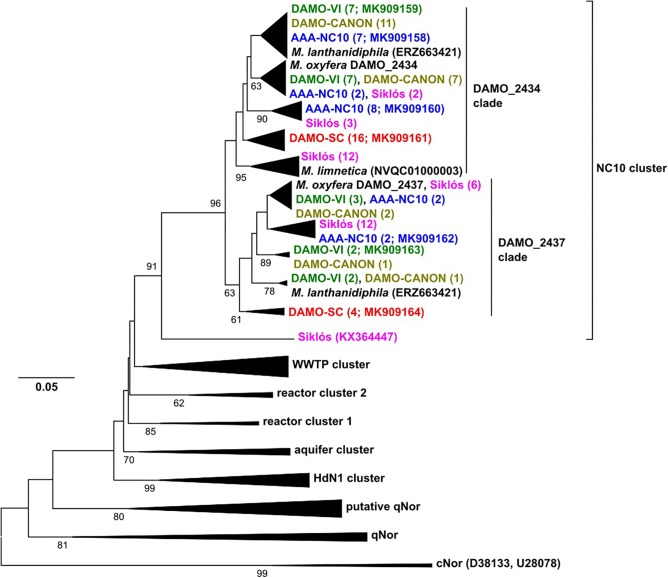
The *nod* gene phylogeny of NC10 bacteria in four reactors and Siklós aquifer. Sequences obtained from different samples are labeled in different color. Sequences from different samples are labeled in different colors and are consistent in all figures. The number in bracket indicates number of clones in the branch or accession number of the reference and representative sequence.

We recovered NC10-related marker genes from four enrichment cultures in chemostats (Table 1) that were active in methane and/or ammonia removal when samples were taken. No more than three different NC10 species-level OTUs were found in each reactor, with one OTU dominating over the others. This was inferred consistently via 16S rRNA, *nod* and *pmoA* gene diversities (Figure 1–3). The low diversity and uneven abundance of NC10 OTUs in each reactor allowed us to compare the phylogenies of 16S rRNA, *nod* and *pmoA* marker genes between reactors.

Phylogeny of 16S rRNA genes showed that in each of the four enrichment cultures, more than one NC10 species/subspecies-level OTU was detected. Five clones (out of 22) of enrichment DAMO-VI and two clones (out of 21) of DAMO-CANON cultures were highly similar (>99%) to the 16S rRNA gene of *M. oxyfera*, indicating these sequences belonged to *M. oxyfera* in a strict sense. Together with the recently sequenced *M. lanthanidiphila* (Versantvoort et al., 2018), the majority of DAMO-VI and DAMO-CANON reactor clones, and some clones from AAA-NC10 and DAMO-SC reactors formed a cluster distinct (97.5% identity) to *M. oxyfera*. The DAMO-CANON reactor had the same dominant NC10 species and a similar NC10 community composition as the DAMO-VI reactor (Figure 1). As the CANON reactor was inoculated with DAMO-VI and anammox biomass (Luesken et al., 2011a), this finding indicated that the co-culturing with anammox bacteria and the constant supply of ammonia did not induce a shift in NC10 population.

In DAMO-SC, nearly all NC10 16S rRNA sequences were closely related to that of *M. sinica* (KU891931 and KT443985), which has ∼97% sequence similarity to *M. oxyfera*. *M. sinica*, originally enriched from paddy soil, are reported as cocci (He et al., 2016), clearly distinct to the morphology of *M. oxyfera*. The dominant NC10 16S rRNA gene sequences in the AAA-NC10 reactor were very similar to the DQ369742 sequence, reported for the first enrichment culture of denitrifying anaerobic methane oxidizers (Raghoebarsing et al., 2006). Nitrate was the sole electron acceptor in the AAA-NC10 reactor, with only low (*ca*. 100 μM) transient nitrite accumulation (Ettwig et al., 2016). Similarly, the initial culture reported by Raghoebarsing et al. (2006) was obtained under high nitrate (>3 mM) and low nitrite (*ca*. 200 μM) availability. These conditions differed from high nitrite concentrations in the DAMO-VI and DAMO-CANON reactors (Luesken et al., 2011a). This suggests that nitrite concentrations could possibly play a role in selecting different NC10 strains. Thus, in addition to exploring diverse environments, varying nitrite and nitrate concentrations during enrichment may also help to cultivate divergent NC10 bacteria.

Interestingly, the dominant NC10 methanotroph *M. limnetica* in lake Zug (Graf et al., 2018) is closely related to lake Constance clones (Figure 1), suggesting some common lake conditions may favor this NC10 species over others. NC10 16S rRNA sequences obtained from the Siklós aquifer were all part of group B of NC10 bacteria (Figure 1). Although NC10 group B bacteria sequences were retrieved from different environments (e.g., Shen et al., 2016), they have not been enriched or isolated thus far, and their ecophysiology is unknown. Environments with only one dominant phylogroup of NC10 sequence types like the Siklós aquifer can be interesting for further elucidating their ecological functions.

### *pmoA* Diversity in Enrichment Cultures and Aquifer Sediment

*pmoA* genes of the NC10 phylum have been previously reported to form a cluster distinct to other aerobic methanotrophs, and have been used to detect NC10 methanotrophs in different ecosystems (Luesken et al., 2011c). In our reactor samples, the NC10 community revealed via *pmoA* was largely consistent to that inferred via 16S rRNA phylogeny, although *pmoA* sequences from the DAMO-SC reactor, instead of the Siklós aquifer, were placed in the basal region of the NC10 *pmoA* cluster. There were at least two different groups of *pmoA* sequences recovered from each reactor, again suggesting multiple NC10 (sub)species to exist in each community. Similar to 16S phylogeny, together with *M. lanthanidiphila pmoA*, the dominant *pmoA* type from the DAMO-VI and DAMO-CANON reactors, as well as five clones from AAA-NC10 reactor, clustered closely together (Figure 2). A minority of clones from the DAMO-VI and DAMO-CANON reactors clustered closely with *M. oxyfera pmoA*. This was congruent with 16S rRNA gene phylogeny (Figure 1). In contrast, *pmoA* from the DAMO-SC reactor and some of AAA-NC10 reactor clones formed distinct clusters (Figure 2). In 16S rRNA phylogeny, the branching of AAA-NC10 clones was deeper than that of DAMO-SC clones, while DAMO-SC clones branched close to the root in *pmoA* phylogeny.

Notably, although nearly all Siklós aquifer 16S rRNA sequences were highly similar and belonged to the group b of NC10 bacteria, Siklós *pmoA* seemed to be more diverse and dispersed among environmental and reactor *pmoA* sequences (Figure 2). Nevertheless, the majority of Siklós *pmoA* sequences formed a separate cluster and were close to NC10 *pmoA* sequences retrieved from Lake Constance (Deutzmann and Schink, 2011) and *M. limnetica*. This could indicate that group b NC10 bacteria are possibly capable of methane driven denitrification as well.

Primers (Table 2) used to recover *pmoA* gene fragments were initially intended to be NC10-specific (Luesken et al., 2011c). However, in reactor DAMO-SC, about 10% of the obtained sequences were affiliated to comammox *amoA* genes, which typically showed 79 and 90% sequence similarity on DNA and amino acid level, respectively, to that of *Nitrospira inopinata* (Daims et al., 2015). Comammox-related *amoA* sequences were not detected in other reactors and aquifer samples, though the same primers were applied. This indicates that the current NC10-specific *pmoA* primers may still need to be improved in specificity, as comammox bacteria are being discovered from more and more different environments (e.g., Xia et al., 2018). The competition of oxygenic denitrifiers and comammox bacteria for nitrite certainly warrants future investigations.

### *nod* Gene Diversity in NC10 Enrichment Cultures and Aquifer Sediment

Besides sequenced NC10 bacteria genomes, environmental *nod* sequences have previously been reported from marine oxygen minimum zone (OMZ), aquifers, wastewater treatment systems, alpine wetland, and microaerobic pollutant-degrading sediment (Padilla et al., 2016; Zhu et al., 2017; Bradford et al., 2018; Zhang et al., 2018). Recently, *nor* sequences with *nod* characteristics were found in the genome of foraminifera, which, however, produced N_2_O (Woehle et al., 2018). In addition to the NC10 cluster, several unidentified *nod* clusters have also been reported from aquifer and wastewater systems (Zhu et al., 2017). Bhattacharjee et al. (2016) obtained several nearly identical *nod* sequences from a NC10 enrichment, although diverse NC10 bacteria were present in the culture.

The reactor *nod* gene sequences retrieved from the four reactors and Siklós aquifer expanded the known diversity of *nod* genes within the NC10 cluster. Compared to 16S rRNA and *pmoA* genes, the *nod* sequences in each reactor were more widely branching, suggesting that *nod* genes were less conserved than 16S rRNA and *pmoA* genes (Figure 3). The overall diversity of *nod* genes was higher than that of 16S rRNA and *pmoA* genes of the NC10 phylum. However, considering that the *Methylomirabilis* species sequenced so far host two distinct *nod* genes (Ettwig et al., 2010, 2012; Graf et al., 2018; Versantvoort et al., 2018), the diversity of NC10 bacteria inferred via *nod* gene was again similar to that of 16S rRNA and *pmoA* genes (Figure 3). Phylogenetically, NC10 cluster *nod* sequences can be divided into two major subgroups, named according to the *nod* homolog of *M. oxyfera* (DAMO_2434 or DAMO_2437) to which they are more closely related (Figure 3). The two *nod* sequences of *M. lanthanidiphila* fell into their respective clades. However, both of the *M. limnetica nod* copies clustered with some Siklós *nod* sequences, and were placed in the root of the DAMO_2434 clade.

The phylogenetic placements of *nod* genes within the DAMO_2434 clade was in good agreement with the phylogeny of NC10 16S rRNA genes: most sequences from DAMO-VI, DAMO-CANON and AAA-NC10 reactors were closely related, while sequences of the DAMO-SC reactor and Siklós were more deeply rooted. This general pattern was also seen amongst the sequences found within the DAMO-2437 clade, except Siküs *nod* sequences were clustered to DAMO-VI and AAA-NC10 reactor sequences, instead of in the basal region (Figure 3). Similar to *pmoA*, Siklós *nod* sequences also represented a higher NC10 diversity than Siklós 16S rRNA genes.

### Comparison of NC10 Abundance via 16S rRNA and *nod* Genes

The general congruence among NC10 16S rRNA, *pmoA* and *nod* gene phylogenies suggested that *nod* can be used as a phylogenetic marker for these oxygenic methanotrophs. In order to validate if the abundance of methanotrophic NC10 bacteria in the environment can be directly quantified by targeting *nod* genes, a new qPCR primer set specific for NC10 *nod*-genes was developed and verified (Table 2).

**FIGURE 4:**
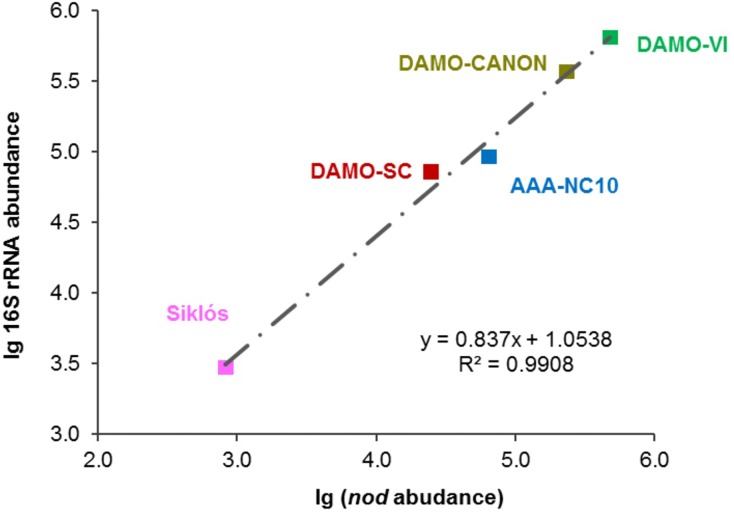
Strong linear relationship between 16S rRNA and *nod* gene abundances observed in these bioreactor and aquifer samples.

In addition to the four reactor cultures, the primer pair was also applied to the Siklós aquifer sediment. Overall, the NC10 methanotroph abundance inferred from *nod* genes showed a strong linear correlation (*R*^2^ > 0.99) with that inferred from NC10 16S rRNA in all samples (Figure 4). The abundance of NC10 bacteria in all samples as quantified via *nod* genes was always lower than their abundance inferred from 16S rRNA genes. For the Siklós aquifer sediment, where NC10 bacteria were not enriched like in the reactor samples, the largest difference was observed: 3.0 × 10^3^ 16S rRNA and 8.3 × 10^2^ *nod* (copy ng^-1^ DNA), but the difference was still within one order of magnitude (Table 1). This might suggest that not all NC10 bacteria are oxygenic denitrifiers. In fact, a methylotrophic member of NC10 without *pmoA* and *nod* genes was recently reported (Hug et al., 2016). Additionally, the difference may also be attributed to different PCR efficiencies when targeting the different genes. These results suggested that it is possible to directly quantify the abundance of oxygenic NC10 populations in enriched cultures as well as in environmental samples by using NC10-specific *nod* gene primers.

## Conclusion

Highly diverse environmental *nod* gene pools in aquatic systems have suggested a high diversity of oxygenic denitrifiers (Zhu et al., 2017), among which NC10 bacteria use methane to drive oxygenic denitrification. Currently, the diversity and ecological importance of NC10 bacteria is not yet well understood. Here, we provide an integrated comparison of the detectability of NC10 populations using three different marker gene systems, and we found that the phylogeny of NC10 *nod* genes was generally congruent with those of 16S rRNA and *pmoA* genes. qPCR analysis targeting 16S rRNA and *nod* genes also resulted in different but still comparable NC10 bacteria abundance. Overall, the results suggest that *nod* can be used as a functional and phylogenetic marker for oxygenic NC10 methanotrophs. In future studies, researchers are suggested to include analyzing *nod* genes to better estimate the ecological importance of oxygenic NC10 bacteria in global methane cycling.

## Data Availability

The datasets generated for this study can be found in the NCBI, MK909154 – MK909164.

## Author Contributions

BZ and TL designed the project. BZ, JW, and LB performed the experiment. BZ analyzed the data with help from KE, BH, and TL. BZ wrote the manuscript with inputs from all authors.

## Conflict of Interest Statement

The authors declare that the research was conducted in the absence of any commercial or financial relationships that could be construed as a potential conflict of interest.
